# The counterintuitive implications of superspreading diseases

**DOI:** 10.1038/s41467-023-42612-9

**Published:** 2023-10-31

**Authors:** Bjarke Frost Nielsen, Kim Sneppen, Lone Simonsen

**Affiliations:** 1 PandemiX Center of Excellence, Roskilde University, Roskilde, Denmark; 2 High Meadows Environmental Institute, Princeton University, Princeton, NJ USA; 3 The Niels Bohr Institute, University of Copenhagen, Copenhagen, Denmark

**Keywords:** Epidemiology, Epidemiology

## Abstract

The superspreading that characterized SARS and now COVID-19 can be rapidly quantified; however, its implications for outbreak control were never well understood. Recent studies point to its profound impact on outbreak dynamics and prospects for effective control of a future Disease X. These insights necessitate research into the mechanisms, impact and different modes of superspreading more widely.

## Teaser

Superspreading is known to have played an important role in the transmission dynamics of SARS-CoV-2. In this Comment, the authors discuss how knowledge of the extent and cause of superspreading is important for designing appropriate control measures for emerging infectious diseases.

As SARS-CoV-2 swept the globe, two major features of its transmission became apparent: its tendency towards superspreading and its airborne nature. In fact, the superspreading tendency was established early on, even before COVID-19 was declared a pandemic in March 2020^1^. The *dispersion parameter k*, which quantifies the degree of heterogeneity in transmission^2^, was determined to be in the range of 0.1–0.2, indicating that a minority (10–20%) of SARS-CoV-2 infected individuals accounted for the majority (80%) of new infections^3^. The numerous well-documented superspreading events where single individuals infected dozens of others without close contact should have been a red flag, strongly suggesting spread by aerosols. The realization that COVID-19 transmission was characterized by airborne transmission was only acknowledged by WHO and the CDC much later, in mid-to-late 2021.

Influenza, the main culprit during the last 150 years of respiratory pandemics, does not exhibit a strong superspreading potential and, while data is scarce, values of *k* close to or exceeding 1 have generally been reported^4,5^. For coronaviruses, the superspreading characteristic was already noted during the 2003 SARS outbreak where the need for a theoretical understanding of its implications for outbreak control was noted^6^. The spread of SARS involved several nosocomial superspreading events as well as an iconic outbreak in a private apartment complex in Hong Kong attributed to spread via aerosols suspended in the building’s plumbing^7^. However, a thorough theoretical understanding of how superspreading impacts mitigation did not arise and interest seemingly faded after the outbreak was contained later that year. MERS, a looming threat since 2012, was also characterized by superspreading, with notable nosocomial superspreading events in South Korea in 2015; again, few theoretical insights were gained about the phenomenon. In the early months of the COVID-19 pandemic, epidemiological evidence of superspreading began piling up once again, reaffirming the need for a theoretical understanding of its mechanisms and implications for outbreak control.

**Fig. 1:**
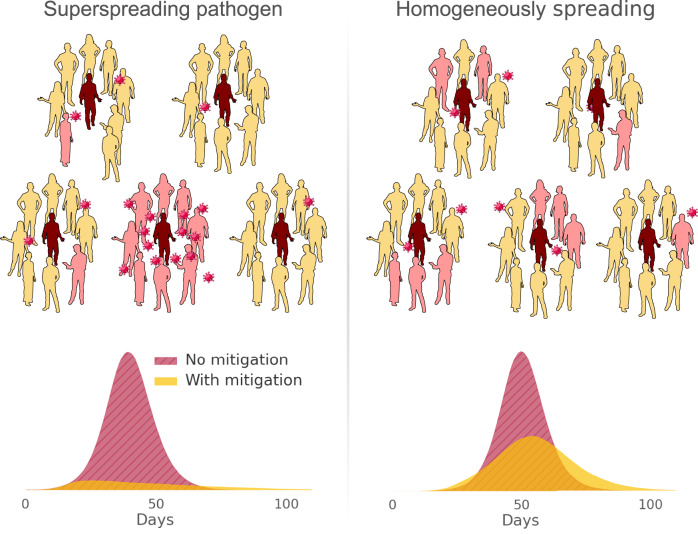
Superspreading as a determinant of outbreak control. Superspreading diseases exhibit a skewed distribution of infectiousness, where most infected individuals transmit to very few or none at all, while a minority spread the disease to large numbers of people (left panel). In this case, limiting gatherings and situations where many people meet (even briefly) has an outsized mitigating effect, as shown by the epidemic curves at the bottom of the left panel, which result from agent-based simulations of a superspreading disease. In diseases where transmission is more evenly distributed (non-superspreading, right panel), such interventions do not hold the same potential for outbreak control. Data from Sneppen et al.^8^. ProPublica’s *Wee People* font used for human silhouettes.

Our recent theoretical studies have shown that superspreading plays a profound role in determining the most effective strategies to curb a disease outbreak. At the same average transmissibility (same *R*_*0*_), an outbreak of a more superspreading disease can be more readily controlled^8^, as illustrated in Fig. 1. Specifically, we found that mitigation strategies that reduce contacts in public spaces are vastly more effective when superspreading is a key driver of high average transmissibility (*R*_*0*_). Consequently, the closing of venues such as concert halls and bars can significantly curb the spread of a superspreading respiratory pathogen, while the same measures may do little to halt a non-superspreading pathogen with the same basic reproductive number. The origin of this effect lies in the statistics of superspreading, which somewhat counterintuitively imply that most infected individuals do not become very infectious. Thus, mitigation of a superspreading disease relies on either a) directly targeting interventions at superspreaders or b) limiting the number of contacts that the typical infected individual has. The former strategy depends on being able to a priori identify the superspreader—often practically impossible—while the latter does not. Model simulations confirm this logic, showing that effective mitigation of a superspreading disease can be achieved by moderate interventions, such as the closure of large events. No such effect was seen for this scenario with an otherwise comparable disease with homogeneous transmission. Thus, it appears that superspreading is an Achilles’ heel for a pathogen like SARS-CoV-2. The transmission statistics of superspreading can be leveraged to drastically improve the effectiveness of contact tracing programmes as well^9^.

Crucially, the interactions between superspreading and mitigation strategies are not readily captured by typical compartmental transmission models, necessitating the use of e.g. agent based models. The limitations of traditional compartment models – and some network models – have also fueled the misconception that the impact of superspreading is largely confined to the early stages of an epidemic. One viewpoint is that superspreading events merely serve as stochastic “sparks” that ignite the initial outbreak, but whose influence is quickly drowned out as the incidence increases. This has been shown not to be the case – when imperfect social mixing is taken into account, it becomes clear that superspreading has a significant impact throughout an epidemic^8,10^.

Adding another layer of complexity is the commonly held belief that superspreaders fuel the rapid initial growth of an outbreak but are subsequently ‘depleted’, leading to a decline in the growth rate. This belief often rests on the assumption that superspreading is a function of hypersocial individuals who both spread and contract the infection at higher rates^11^. While this pattern describes some instances of superspreading well, such as the recent mpox outbreak^12^ and some sexually transmitted infections (related to the concept of *Core Groups*^13^), it does not apply universally. Particularly for diseases where superspreading has a biological basis – rendering some individuals inherently more infectious – the picture changes. In this case, superspreaders are not necessarily ‘super-receivers’, and their influence may persist throughout the outbreak^10^. In other words, the impact of superspreading depends strongly on the degree of correlation between susceptibility and infectiousness. Therefore, it is critical to distinguish between superspreading as a consequence of social behavior/contact rate heterogeneity, and superspreading rooted in biological factors, all of which may affect susceptibility as well as infectiousness.

At present, the exact etiology of the superspreading phenomenon remains incompletely understood. The fact that some respiratory pathogens are highly superspreading, while others are not, likely depends on several behavioral as well biological factors, including aerosolized spread, the degree of pre- and asymptomatic transmission, the minimal infective dose, as well as person-to-person variability in respiratory viral load and aerosolization^5,14^. Infections that have a high degree of pre- and asymptomatic transmission, and those with a low minimum infective dose, are likely to have a greater superspreading potential; asymptomatic transmission increases the risk of (unknowingly) infectious individuals participating in public life, while a low minimum infective dose increases the range of transmission and decreases the necessary exposure time. High person-to-person variability in respiratory viral load is another factor that may contribute significantly to superspreading. In infections such as COVID-19, where viral loads vary enormously from person to person, a pronounced degree of superspreading is observed, pointing to superspreading as an intrinsic feature of the pathogen and its interaction with the human host^5,15^. Understanding these factors is crucial in developing effective mitigation and containment strategies for future pandemics. Thus, there is a need for research into the mechanisms behind superspreading, and potentially to differentiate between different types of heterogeneous spread.

Even in the absence of a clear etiology, our research shows that knowledge of the bare transmission statistics is a powerful thing which can enable the design of improved control strategies. In general, this highlights the importance of getting the intricacies of transmission right when the next pandemic threat emerges. COVID-19 has shown that the dispersion factor *k* can be rapidly ascertained for an emerging pathogen. This suggests that *k* should join the basic reproductive number (*R*_*0*_), the generation time and the infection and case fatality ratios (IFR and CFR) as critical parameters needed for initial response to an emerging pandemic. Crucially, knowing *k* can not only aid the design of interventions but may also provide early indications of airborne transmission potential. However, this rapid quantification of superspreading depends on the timely availability of high-quality data, including detailed low-bias spatiotemporal incidence data and representative viral sequencing, highlighting the importance of rapidly scalable epidemiological and genomic surveillance.

With pandemic preparedness historically geared towards influenza A, we had not anticipated a superspreading disease like COVID-19, nor were the implications for control understood. Coronaviruses, however, have repeatedly emerged as significant pandemic threats over the last two decades, each one exhibiting considerable superspreading potential. As we move forward, it is crucial that mathematical models of *Disease X* scenarios take this phenomenon into account.
